# A Narrative Review: A1 and A2 Milk Beta Caseins Effect on Gut Microbiota

**DOI:** 10.3390/nu18010138

**Published:** 2026-01-01

**Authors:** Sathya Sujani, Klaudia J. Czerwinski, Dennis A. Savaiano

**Affiliations:** Department of Nutrition Science, Purdue University, West Lafayette, IN 47906, USA; simaduwa@purdue.edu (S.S.); kczerwi@purdue.edu (K.J.C.)

**Keywords:** beta casomorphin-7, dysbiosis, milk protein, personalized nutrition, short chain fatty acids

## Abstract

**Background/Objectives:** The composition and function of gut microbiome is significantly influenced by dietary factors. Growing evidence suggests that A1-type and A2-type beta casein (β-CN) may exert distinct effects on the gut environment, with implications for digestive discomfort and broader health outcomes. This review summarizes current evidence on how milk-derived A1 and A2 β-CN affect the gut microbiota. **Methods:** We conducted a literature search using PubMed, Web of Science, and Scopus to identify studies examining effects of milk β-CN on gut microbiota. **Results:** A total of eight studies were included. Results show inconsistencies within the limited number of studies. However, compared to A2, A1 β-CN was more frequently associated with dysbiosis and an increased abundance of potentially pathogenic species. Conversely, A2 β-CN promoted microbial diversity, which is linked to improved gut integrity and metabolic health. **Conclusions:** These findings suggest that β-CN variants distinctly influence the gut microbiota composition, and results were more significant in immunosuppressed subjects or those with other underlying health conditions, indicating that dairy products rich in A2 β-CN may offer advantages in personalized dietary management. However, well-designed human studies are essential to translate findings from rodent models to clinically relevant outcomes and future research should focus on mechanistic exploration and population-specific responses.

## 1. Introduction

Cow’s milk has long been a dietary staple in many cultures, providing essential nutrients and contributing to overall health and well-being [1,2]. However, some individuals experience digestive discomfort after milk consumption, often attributed to lactose intolerance [3,4]. Notably, the 2010 National Institutes of Health consensus statement on “lactose intolerance and health” reports that many individuals who self-report lactose intolerance are not diagnosed with lactose malabsorption, suggesting that the source of digestive discomfort may not be directly related to lactose [5]. Milk proteins, specifically different types of beta caseins (β-CNs) have emerged as a potential cause of digestive discomfort, offering an alternative hypothesis for milk intolerance [6,7].

Milk proteins are primarily categorized into two groups: CNs and whey proteins [8]. Caseins account for about 80% of protein content in milk and exist in four isoforms. The most common isoform is β-CN and up to date at least 15 genetic variants of β-CN have been identified [8,9]. A1 type and A2 type are the primary variants in commercially produced milk and appear to cause different physiological effects when consumed [8,10]. Milk produced in USA, New Zealand, and Australia also contains less common variants of β-CN, which are A3, B, C, and I [11,12]. Variants B and C have a similar amino acid structure to A1 while the amino acid structure of A3 and I are similar to A2 [13]. The distribution of A1 and A2 β-CN variants varies by cattle breed and geographic origin. A1 β-CN is more prevalent in dairy cattle of Northern European origin, such as Friesian and Ayrshire breeds, whereas A2 β-CN is more commonly found in breeds, including but not limited to Guernsey, Jersey, and native Indian cattle [14,15]. The structural difference between A1 and A2 arose from a single nucleotide polymorphism in “new world” cows; an amino acid mutation at 67th position, which replaced a proline in the historical ‘old world’ A2 β-CN with a histidine in “new world” A1 β-CN [16]. Due to the presence of histidine, human gastrointestinal enzymes, and the microbial proteolytic system can cleave A1 β-CN and release a bioactive peptide (beta-casomorphin-7, BCM-7) during digestion. BCM-7 has been implicated in various health issues, particularly those related to the gastrointestinal system [17,18]. Conversely, digestion of the A2 β-CN does not generate BCM-7, [19]. Impacts of BCM-7 on digestive discomfort may be multi-faceted including increased gastrointestinal transit time, increased inflammation, and alterations in the gut microbiota composition [20].

The human gut microbiome plays a critical role in maintaining health, helping to regulate digestion, metabolism, and immune responses [21,22]. Diet is a significant factor influencing the composition and function of the gut microbiota. Emerging evidence suggests that A1 and A2 β-CN may differently affect the gut environment, may alter digestive metabolites (especially short chain fatty acids, SCFA) and lead to digestive discomfort and other health issues [17,23,24]. Research in this area is still in early stages, and most researchers have used animal models and reported inconsistent results. The research on β-CNs, particularly differential effects of A1 and A2 in health and moreover its effect on gut microbiota has gained attention recently following the commercialization of A2 β-CN-rich milk. The objective of this review is to summarize current evidence regarding the effects of A1 and A2 β-CN on the gut microbiota and potential health implications.

## 2. Materials and Methods

**Figure 1:**
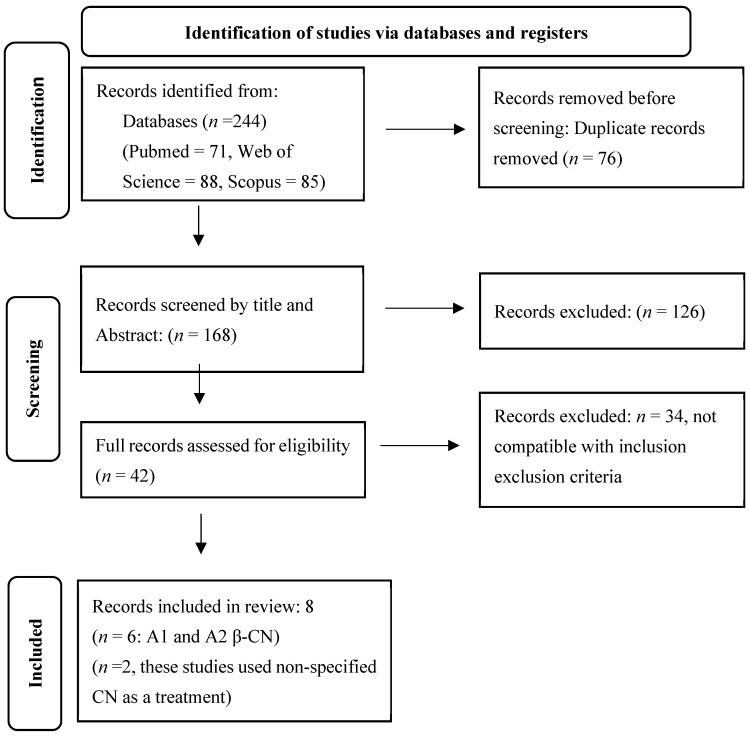
PRISMA flow chart.

We conducted a comprehensive search for original research articles in PubMed, Web of Science, and Scopus, from 8 November 2024 to 10 May 2025. The following keywords were used: (“CN” OR “A1 β-CN” OR “A2 β-CN” OR “β-CN”) AND (dairy OR milk OR “cow’s milk”) AND (“gut microbiome” OR “gut microbiota” OR “intestinal microbiota”) AND (human OR rat OR mice OR murine). Two authors independently performed the literature search. Figure 1 depicts the PRISMA flow diagram for this study.

The inclusion criteria were original research articles published in the English language, full-text accessibility, use of dairy-derived β-CN as an intervention in human or rodent models, and analysis of gut microbiota (microbiota analysis of fecal or samples from any part of the small intestine was considered) via 16S rRNA sequencing or metagenomics methodologies. Studies that did not include gut microbiota analysis were excluded. The year of publication was not restricted and studies published from 2018 to 2023 were qualified. Methodological rigor was ensured by restricting inclusion to peer reviewed, full text original studies and excluding conference abstracts, theses, preprints, and any form of unpublished or non-peer reviewed literature.

## 3. Results

### 3.1. Study Characteristics

Our search in Pubmed, Web of Science, and Scopus yielded 71, 88, and 85 articles, respectively. After removing 76 duplicates, 168 publications were screened by title and abstract, and 126 articles were removed. The full texts of the remaining 42 articles were reviewed for eligibility. Of these, eight articles met the inclusion criteria and were included in the review. Out of eight, six studies investigated differential effects of A1 and A2 β-CN while two studies used unspecified β-CN as intervention. Seven studies used murine models and one studied human subjects. Seven studies employed 16S rRNA sequencing while one study used metagenome shotgun pyrosequencing.

### 3.2. A1 and A2 β-CN and Gut Microbiota

The summary of the eight selected studies is presented in Table 1. To enhance clarity and facilitate interpretation, the findings are organized according to taxonomic order.

#### 3.2.1. Phylum Level

Changes at the phylum level in response to β-CN variants were inconsistent across studies. In human interventions, A1 β-CN was associated with reductions in Firmicutes and Bacteroidetes, whereas A2 β-CN enriched Actinobacteria [27]. In animal models, A2 β-CN increased Proteobacteria, while studies of β-CN supplementation without specification of variant also reported enrichment of both Proteobacteria and Bacteroidetes [29]. Collectively, these findings suggest that phylum level changes are less consistent compared to those observed at deeper taxonomic levels.

#### 3.2.2. Family Level

Noticeable differences were detected at the family level. Guantario et al. [25] showed predominance of Deferribacteraceae and Desulfovibrionaceae in the A2 group, whereas Ruminococcaceae was enriched in A1-fed mice. In the study by Nuomin et al. [24], Muribaculaceae and Desulfovibrionaceae were significantly higher in A1-fed mice compared to A2, while Enterobacteriaceae and Enterococcaceae dominated in the control group. Human data from Song et al. [27] indicated enrichment of Lachnospiraceae, Bifidobacteriaceae, and Coriobacteriaceae with A2 milk consumption, while Enterobacteriaceae were increased in the A1 group. In addition, across interventions, Lactobacillaceae and Clostridiaceae remained high in abundance, while Akkermansiaceae, Bacteroidaceae, and Rikenellaceae were consistently reduced [24].

#### 3.2.3. Genus Level

At the genus level, A2 β-CN was generally associated with enrichment of beneficial taxa. Song et al. [27] observed increases in Bifidobacterium and Blautia in humans following A2 milk intervention, while Li et al. [18] reported increases in Lactobacillus in immunocompromised mice. In contrast, Liu et al. [29] found elevated Escherichia in β-CN supplemented groups, and Zhang et al. [28] reported higher abundances of Alistipes, Odoribacter, Blautia, and Lachnospiraceae NK4A136 group. Decreases in Prevotellaceae related groups were also noted with β-CN supplementation [28]. These results indicate that genera promoted by A2 β-CN are generally associated with probiotic or commensal functions.

#### 3.2.4. Species Level

Differences at the species level were most pronounced. Chia et al. [26] reported that A1 β-CN increased *Streptococcus pyogenes* and *Streptococcus suis* and reduced *Enterobacter cloacae*, *Enterobacter hormaechei*, and *Klebsiella oxytoca*. Liu et al. [17] observed that *Lactobacillus animalis* was significantly enriched in the A2 group, while Li et al. [18] further confirmed increases in *Lactobacillus* species in immunocompromised mice fed A2 milk. In human interventions, Song et al. [27] demonstrated that A2 milk increased *Bifidobacterium longum* and *Blautia* species. In contrast, Liu et al. [29] showed that β-CN supplementation led to higher levels of *Candidatus Saccharibacte* and *Escherichia*. Taken together, A2 β-CN appears to enhance beneficial species, whereas A1 β-CN promotes enrichment of species with pathogenic potential.

In summary, phylum level differences remain inconsistent, but evidence at the family, genus, and species levels indicate clearer trends. For an example, Gunatario et al. [25] observed enrichment of *Deferribacteraceae* and *Desulfovibrionaceae* following A2 β-CN intervention, whereas Nuomin et al. [24] reported increased *Desulfovibrionaceae* and *Muribaculaceae* in response to A1 β-CN. Despite these inconsistencies, A2 β-CN is generally associated with greater microbial diversity and the enrichment of beneficial taxa such as *Lactobacillus animalis* and *Bifidobacterium longum*, whereas A1 β-CN tends to promote dysbiosis through increases in *Escherichia* and potentially pathogenic *streptococci*. These findings suggest that A2 β-CN may contribute to a more favorable gut environment compared to A1.

#### 3.2.5. Short Chain Fatty Acid (SCFA) Profile and Gut Microbiota

Four studies reported SCFA in response to different β-CN [17,18,24,25]. While two studies reported no differences in SCFA, Li et al. [18] reported a significant increase in acetate, propionate, butyrate, and total SCFA in the A2-treated group. A three-fold increase in isobutyrate in both A1 and A2 groups compared to the control group was reported by Guantario et al. [25]. The inconsistent and limited results suggest the need for additional evaluation of SCFAs in response to different β-CNs. Perhaps the effect is simply related to the degree of fermentation happening. Strict controls are needed to better understand if differential fermentation actually occurs based on β-CNs.

## 4. Discussion

This narrative review summarizes the available research on the effects of milk A1 and A2 β-CN protein on gut microbiota alterations, SCFAs, and plausible impacts on health. The gut microbiota was differentially modulated in response to CN interventions. Previous studies have shown that CN supplementation can increase the Firmicutes/Bacteroidetes (F/B) ratio, a microbial shift often associated with obesity and diabetes [30,31]. However, the studies included in this review did not report an elevated F/B ratio in response to either A1 or A2 β-CN. In contrast, the only human study included in this work reported that A1 β-CN significantly reduced the abundance of both Firmicutes and Bacteroidetes [27]. Previous studies that reported independent increases in Firmicutes and decreases in Bacteroidetes have been linked to weight gain, particularly in infants and children [32,33,34,35]. However, Firmicutes represent a highly heterogeneous phylum with diverse metabolic roles, limiting broad functional interpretation based on abundance alone. Several studies we included in this review reported high levels of Firmicutes in response to both A1 and A2 interventions in mouse models making it challenging to draw solid conclusions [24,25]. Notably, A1 β-CN was associated with a greater increase in Firmicutes. The greater increase in Firmicutes observed with A1 β-CN may warrant further investigation, as enrichment of Firmicutes has been hypothesized but not established to contribute to metabolic dysregulation through microbiota modulation. While A1 is associated with a greater relative abundance of Firmicutes, A2 β-CN promoted a more diverse phylum profile including Firmicutes, Deferribacteres, Proteobacteria, and Actinomycetota suggesting a potentially balanced and metabolically favorable gut environment rather than an isolated increase in a single phylum. Whether the broader microbial diversity promoted by A2 β-CN reflects a more metabolically favorable gut environment compared to A1 β-CN, which remains an open hypothesis to explore in different health contexts. Additionally, Desulfovibrionaceae which is associated with low BMI was reported to be distinctive in both non-specific CN and A2 β-CN interventions suggesting potential benefits of milk CN on weight management [25,32].

The abundance of Lactobacillus showed inconsistency with CN supplementation. However, A2 β-CN interventions resulted in higher abundance of *Lactobacillus* spp. in comparison to A1 β-CN [17,18]. Lactobacillus is known for its capacity to strengthen the intestinal barrier via increased mucus production, stimulating the release of anti-microbial peptides, increased production of immunoglobulin A, and improved tight junction integrity [36]. The Liu et al. [17] study also reported increased villus height and crypt depth of the duodenum in the A2 β-CN group. Determining whether the observed improvements in duodenal morphology are linked to shifts in Lactobacillus abundance, or instead arise from broader microbial community changes, warrants further investigation.

The abundance of Ruminococcaceae was more significant in response to A2 β-CN compared to A1 β-CN. The Ruminococcaceae family plays an important role in maintaining gut health by producing butyrate and other SCFAs and it also suppress the colonization of bacteria that are generally considered as harmful [37]. Decreased abundance of Ruminococcaceae has been linked with inflammatory bowel diseases, including ulcerative colitis and Crohn’s disease, hepatic encephalopathy, and *Clostridium difficile* infections [38,39,40,41]. A recent study found that a higher relative abundance of Ruminococcus in gut microbiota is linked to reduced cardiovascular risk in obese individuals [42]. The observed increase in Ruminococcus following A2 β-CN intervention raises the possibility that A2-dominant milk could positively influence cardiovascular health by modulating the gut microbiota [18]. Notably, an increased relative abundance of Ruminococcaceae has been reported in the gut microbiota of individuals with neurodegenerative diseases such as Parkinson’s and Alzheimer’s disease [43,44]. However, these correlations should not be interpreted as evidence of a causal relationship. This highlights the complex role of Ruminococcaceae raising the question if its increase following A2 β-CN reflects a net health benefit. At the same time, its reported links to neurodegenerative diseases complicate the interpretation, underscoring the need for future studies to clarify the complex role of Ruminococcaceae in metabolic health and neurological disease risk.

Increased levels of *S. pyogenes* and *S. suis* were reported in NOD/ShiLtJ mice in response to A1 β-CN intervention [26]. Both these Streptococcus species are generally recognized as pathogenic to humans, capable of causing various infections ranging from mild throat infections to bacterial meningitis [45,46]. While the specific impact of *S. pyogenes* and *S. suis* on human gut health from increased abundance remains underexplored, it is important to note that the study used NOD/ShiLtJ mice, a model for type 1 diabetes, a condition known to increase susceptibility to infections [26]. These findings suggest a potential association between A1 β-CN consumption and microbial patterns linked to opportunistic pathogens; however, the evidence remains insufficient to draw conclusions regarding health risks, particularly for individuals with impaired immune function or metabolic disease. A study by Guantario et al. [25] offers a new perspective on the effects of β-CN. They reported a significant increase in the abundance of opportunistic pathogenic bacteria, such as Enterobacteriaceae and Enterococcaceae, in the control group compared to all milk CN supplemented groups. Thus, milk, regardless of its β-CN type, may be beneficial for controlling these pathogenic bacteria.

Song et al. [27] reported an increased abundance of Actinobacteria, particularly Bifidobacterium, after A2 β-CN consumption and the study by Li et al. [18] reported increased abundance of Bifidobacterium in mice in response to A2 β-CN. Bifidobacterium is recognized for its beneficial role in a wide range of health implications including but not limited to improved digestion and immune function [47,48]. These observations imply that A2 β-CN associated increases in Bifidobacterium may represent a recurring pattern across species and experimental models. However, the consistency and underlying mechanisms of this effect require further confirmation. While these findings are promising, additional well-designed human studies are necessary to determine their clinical relevance and to clarify whether such microbial changes can be meaningfully leveraged in personalized or precision nutrition strategies.

Alterations in the gut microbiota changes the profile of SCFAs, the main metabolites produced by the gut microbiota, which are important in gut function, immune regulation and the gut–brain axis [49,50,51]. Notably, Li et al. [18] also observed enrichment of Lactobacillus and more stable intestinal flora in the A2 group, which may associate with the concurrent increase in SCFA production. Positive association between enrichment of Lactobacillus species and increased SCFA production leading to better gut health has been reported in human and animal models [52,53]. Increased total SCFA production in response to A2 milk was also reported in two clinical studies [54,55]. However, because these studies did not explore the corresponding gut microbiota dynamics, it is impossible to establish whether the changes in SCFA levels were due to a microbial-driven effect related to the different β-CN interventions. A three-fold increase in isobutyrate in both A1 and A2 compared to the control group shows that irrespective of the β-CN type the consumption of milk exerts beneficial modulations in gut microbiota [25]. Studies have shown positive health effects of isobutyrate in both murine and human models ranging from resistance to inflammatory bowel disease in pigs and protective effect against intestinal damage likely through microbiota remodeling [56,57]. However, the observed increase in isobutyrate cannot be explained by the microbial shifts reported in this study, as the enrichment of Deferribacteraceae and Desulfovibrionaceae with A2, or Ruminococcaceae with A1, are not taxa typically associated with isobutyrate production. Overall, the current evidence does not support a consistent or genotype specific effect of A2 β-CN on SCFA production, as reported changes appear to be context dependent and limited to specific experimental settings. Consequently, potential links between β-CN variants, gut microbial composition, and SCFA profiles remain speculative and require further investigation.

**Figure 2:**
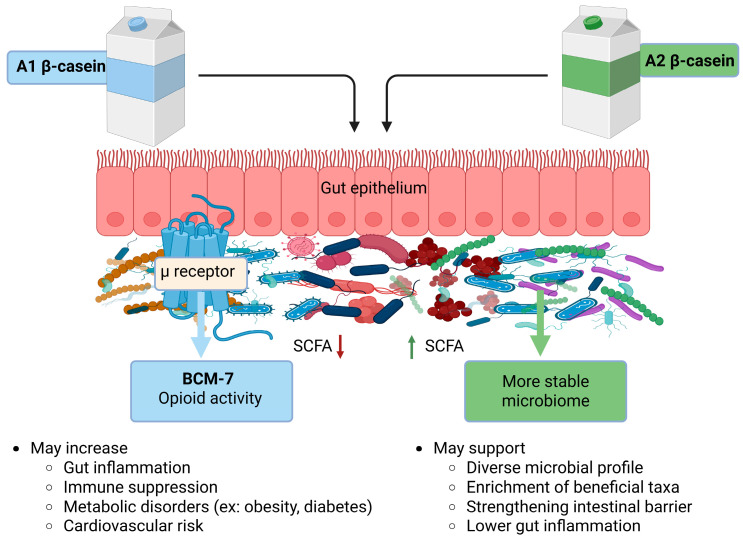
Differential effects of A1 and A2 β-CN on gut microbiota and health implications. SCFA: short chain fatty acid; BCM-7: beta casomorphin-7. ↓ Arrow denotes decreased SCFA and ↑ denotes increased SCFA.

Available evidence suggests that milk-derived β-CN variants exert complex and context dependent effects on the gut microbiota and these effects are summarized in Figure 2. A2 β-CN has been associated with comparatively more favorable gut microbial profiles, including increased microbial diversity and enrichment of taxa generally regarded as beneficial. However, findings across studies remain inconsistent. While these observations indicate that A2 β-CN may hold promise as a dietary component for individuals with gut sensitivity or immune challenges, the current evidence largely derived from animal models limiting definitive conclusions regarding its therapeutic potential in humans.

Given that the primary metabolic distinction between A1 and A2 β-CN lies in the generation of bovine BCM-7, future research should also prioritize direct assessment of bovine BCM-7 activity, as this was not addressed in the studies reviewed here. BCMs are known to bind to µ-opioid receptors in the gastrointestinal tract and central nervous system, with bovine BCM-7 demonstrating particularly high opioid activity [58,59]. Through μ-opioid receptor engagement, BCM-7 has been implicated in delayed gastrointestinal transit, altered intestinal secretion (e.g., increased mucin secretion in response to A1 β-CN), and changes in epithelial barrier function—host-mediated effects that can indirectly shape the gut microbial environment [17,20,60,61]. In addition to opioid-dependent mechanisms, evidence from animal and human studies suggests that BCM-7 may interact with non-opioid pathways, including modulation of glucose transporters such as GLUT2 and GLUT4 and induction of pro-inflammatory, T-cell-mediated immune responses, although the relevance of these pathways to gut microbiota regulation remains unclear [62,63]. Notably, A1 β-CN induced stimulation of dipeptidyl peptidase-4 expression and the presence of BCM-7 in the jejunum further suggests the involvement of opioid-independent mechanisms that warrant further investigation [20]. While several studies reviewed here reported microbiota alterations in response to β-CN variants, it remains uncertain whether these changes are mediated primarily through opioid-dependent or independent pathways; however, current evidence more strongly supports host-mediated, opioid-linked mechanisms rather than direct microbial interactions.

### Limitations and Future Directions

With the exception of a single investigation, research examining the effects of β-CN variants on microbiota has been conducted in animal models. While animal models provide valuable mechanistic insights, substantial biological and functional differences between murine and human gut microbiota, including differences in microbial composition, metabolic capacity, immune interactions, and dietary responsiveness should be considered. These differences profoundly influence host–microbiota interactions and limit direct translational inference from animal models to humans. This further emphasizes that any potential clinical or precision nutrition implications discussed in this review should therefore be considered as preliminary and hypothesis-generating rather than confirmatory. Interpretation of existing data is further complicated by methodological heterogeneity, including differences in study design (e.g., different animal models, intervention doses and timeline, host immune status) and microbiota profiling approaches, as well as the tendency for observed effects to occur at lower taxonomic levels. Future studies incorporating genus, family, and species level analyses may therefore provide a more comprehensive understanding of β-CN-associated microbial changes. Furthermore, mechanistic studies integrating BCM-7 measurements with gut microbial composition and microbial metabolite production, particularly SCFA, are essential to clarify the biological relevance of opioid-dependent pathways and their potential implications for precision nutrition strategies.

## Figures and Tables

**Table 1:** Summary of the papers with β-CN or A1 and A2 β-CN as an intervention.

Reference	Subjects	CN Interventions	Days of CN Intervention	Sequencing Method	Distinctive Changes in Gut Microbiota	
					A1 β-CN	A2 β-CN
[25]	Balb-C Mice	Control A2A2 A1A2	28	16S rRNA sequencing targeting V3–V4 hypervariable region	Ruminococcaceae	Deferribacteraceae, Desulfovibrionaceae
[26]	NOD/ShiLtJArc mice	A1 A2	210	Metagenome shotgun pyrosequencing (paired end with read length of 101 nucleotides)	*Streptococcus pyogenes*, *Streptococcus suis*	Not reported
[17]	Pathogen-free Balb/c mice	Control A1 A2	28	16S rRNA sequencing targeting V3–V4 region	Not reported	Ruminococcaceae, *Lactobacillus animalis*
[24]	C57BL/6 mice	Mixed CN A1 A2 Soy Egg white	28	16S rRNA sequencing targeting V4 region	Desulfobacterota, Muribaculaceae, Staphylococcaceae	Eggerthellaceae, Lachnospiraceae, Enterococcaceae
[18]	Pathogen-free Balb/c mice	Normal Model Low A2 Medium A2 High A2 High beta	35	16S rRNA sequencing targeting V3–V4 region	*Streptococcus*, *Prevotella*, *Thermoactinomyces*, *Anaerotruncus*, *Ethanoligenens*	*Lactobacillus*, *Weissella*, *Ruminococcus*, and *Bifidobacterium*
[27]	Humans	A1 milk A2 milk	35	16S rRNA sequencing targeting V3–V4 region	No changes in alpha diversity Significantly decreased Firmicutes and Bacteroidetes Significantly increased Actinobacteria	No changes in alpha diversity Significant shift in beta diversity between before and after A2 consumption Enrichment of classes Actinobacteria, Coriobacteria and family Lachnospiraceae, Bifidobacteriaceae, and Coriobacteriaceae and genus Bifidobacterium and Blautia, and species *Bifidobacterium longum* and *Blautia wexlerae*
* [28]	BALB/C mice	Control Model Kappa CN (KCN) β-CN βK-CN CN micells	28	16S rRNA sequencing targeting V3–V4 region	Increased *Muribaculaceae*_*norank*, *Lachnospiraceae NK4A136* group, *Lachnospiraceae*_uncultured, Alistipes, Odoribacter, Blautia, and *Clostridia UCG-014_norank* Reduced Prevotellaceae	
* [29]	C57BL/6J male mice	Control Model Positive control β-CN group Bioactive peptide group	7	16S rRNA sequencing targeting V3–V4 region	Greater Shanno index and lower Simpson index Increased Proteobacteria, Bacteroidetes and Candidatus_Saccharibacte Highest level of *Escherichia* Increased Enterobacteriaceae and Erysipelotrichaceae at family level	

* These studies investigated the effect of β-CN without specifying A1 or A2.

## Data Availability

No new data were created or analyzed in this study. Data sharing is not applicable to this article.
